# Hydrogels Activated with Plant Extracts/Bioactive Compounds for Cancer Treatment: From Design to Application

**DOI:** 10.3390/gels12070583

**Published:** 2026-07-02

**Authors:** Sema Nur Belen, Ozgur Ozay

**Affiliations:** Laboratory of Biomaterials Research, Department of Bioengineering, Faculty of Engineering, Çanakkale Onsekiz Mart University, Çanakkale 17100, Türkiye; ozgurozay@comu.edu.tr

**Keywords:** plant extract, plant-derived bioactive compound, hydrogel, cancer treatment, controlled release

## Abstract

Plant extracts and plant-derived bioactive compounds are considered important natural agents in cancer research due to their antiproliferative, pro-apoptotic, antioxidant, anti-inflammatory, and anti-angiogenic effects. However, the low solubility, limited bioavailability, instability, and challenges in their standardization directly limit their therapeutic use. Therefore, the development of new delivery systems has become necessary. In this context, hydrogels are among the biomaterial platforms gaining attention in cancer treatment. This review provides a comprehensive assessment of the potential of hydrogel systems containing plant extracts and plant-derived bioactive compounds in cancer treatment. The article discusses cancer types, the limitations of current treatments, mechanisms of action of plant-derived bioactive compounds against cancer, stimulus-responsive hydrogel systems, and the design criteria for extract-loaded hydrogels. In addition, hydrogel systems containing plant-derived components and combination approaches that use these components alongside anticancer drugs have been investigated. According to the literature, these compounds may increase anticancer activity through local, prolonged release, reduce the toxicity of chemotherapeutic agents in some cases, and exhibit complementary or synergistic antitumor effects with chemotherapeutic drugs. They also point out the potential of treatment strategies targeting the tumor microenvironment. However, researchers need to conduct more comprehensive studies on extraction standardization, biosafety, release kinetics, in vivo efficacy, and clinical scalability. In conclusion, hydrogel systems containing plant extracts and plant-derived bioactive compounds should be considered not as direct alternatives to cancer treatments but as rational biomaterial platforms that enable controlled release, local application, and combination therapies.

## 1. Introduction

Cancer is considered one of the most significant global health issues due to its high incidence and mortality rates. A study conducted in 2022 reported approximately 20 million new cases of cancer and 9.7 million cancer-related deaths [1]. This situation demonstrates that cancer is not merely uncontrolled cell proliferation but rather a complex process involving the interplay of numerous biological processes, such as evasion of apoptosis, angiogenesis, invasion, metastasis, evasion of the immune response, and interaction with the tumor microenvironment [2]. For this reason, it is necessary to improve current cancer treatment methods and to conduct research into more selective, safe, and effective systems [2,3].

Among the studies conducted for this purpose, plant extracts and plant-derived bioactive compounds are also being used as therapeutic agents. The literature indicates that bioactive compounds derived from plants have demonstrated therapeutic effects in inhibiting and halting cancer cell proliferation. They have also been reported to promote apoptosis in cancer cells and to benefit patients by regulating harmful biological processes in the tumor microenvironment [4]. However, there are also pharmaceutical limitations to the therapeutic use of plant extracts. This calls into question systems capable of delivering and releasing these compounds in a controlled manner to more effectively harness the anticancer potential of plant extracts [5,6]. Hydrogels, which are biomaterial-based delivery systems, stand out among the materials considered in this context [7,8].

Hydrogel-based carriers, particularly for cancer treatment, are considered useful platforms because they facilitate the sustained release of anticancer agents at the tumor site [9,10]. This situation offers opportunities for research into pH-, temperature-, light-, enzyme-activity-, or redox-sensitive hydrogel systems, enabling more controlled drug release in the target region as part of more selective delivery strategies in cancer treatment [11,12]. As a result, hydrogel systems are significant for preserving natural bioactive compounds, particularly phytochemicals such as curcumin, which have low solubility and limited bioavailability, within hydrogel matrices and for delivering them more effectively to the tumor site [5,6,9,10,13]. The purpose of this article is to evaluate the potential of hydrogel systems containing plant extracts and plant-derived bioactive compounds in cancer treatment.

In this context, this review integrates the pharmaceutical limitations of plant extracts and plant-derived bioactive compounds with the controlled-release advantages of hydrogel systems within a single framework for cancer treatment.

## 2. Types of Cancer

**Figure 1:**
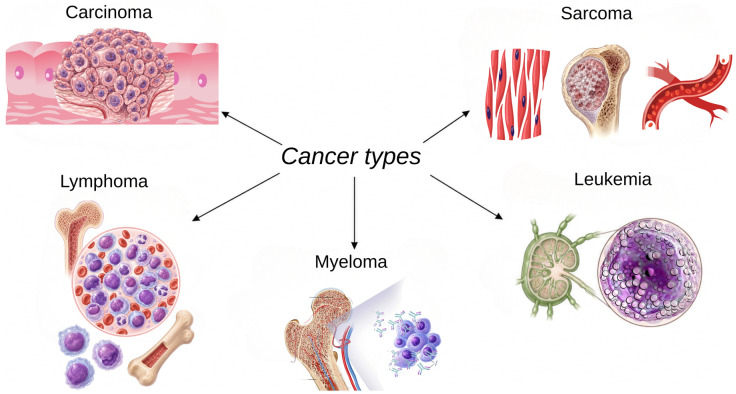
Classification of the principal cancer types according to their tissue of origin. (Carcinomas: epithelial tissues; sarcomas: connective tissues; leukemias: hematopoietic cells; lymphomas: lymphatic system; and myelomas: plasma cells.)

Cancers are generally classified by the cell or tissue type from which they originate (Figure 1). Figure 1 shows the classification of cancer types.

This classification includes carcinomas arising from epithelial tissue, sarcomas arising from connective and supporting tissues, leukemias arising from hematopoietic tissues, lymphomas arising from the lymphatic system, and myelomas arising from plasma cells [14]. This approach provides a fundamental framework for understanding the biological behavior and clinical characteristics of cancer [14,15].

### 2.1. Common Types of Cancer

According to GLOBOCAN 2022 data, the most common cancers are lung cancer, breast cancer, colorectal cancer, prostate cancer, non-melanoma skin cancer, and stomach cancer [1].

Lung cancer is among the most common cancers worldwide. This disease is the leading cause of cancer death, with 2.5 million new cases and 1.8 million deaths [1,16]. Breast cancer is the most common cancer in women. It is estimated to cause 2.3 million new cases and 670,000 deaths [1,17]. Colorectal cancer, which originates in the colon and rectum, is one of the most common cancers in the world. This type of cancer is a leading cause of death, with 1.9 million new cases and more than 900,000 deaths [1,18]. Prostate cancer is one of the most common cancers in men. Every year, about 1.5 million new cases of prostate cancer are found around the world [1,19]. Stomach cancer accounts for about 969,000 new cases and 660,000 deaths [1,20]. It is often diagnosed at advanced stages and is one of the types of cancer with a poor prognosis [21]. Liver cancer (particularly hepatocellular carcinoma) is one of the types of cancer with the highest mortality rates. Research data indicate that it causes about 758,000 deaths [1,22]. Cervical cancer is one of the most common types of cancer among women. It is responsible for about 660,000 new cases and 350,000 deaths. These statistics indicate that cervical cancer is the fourth most common cancer type among women [23]. Ultraviolet (UV) radiation primarily causes non-melanoma skin cancers and melanoma [24,25]. The mortality rates for non-melanoma skin cancers, which are widespread worldwide, are relatively lower compared to those of melanoma. Melanoma, however, is a rare but aggressive form of skin cancer with a high metastatic potential [25].

The types of cancer discussed here account for a significant portion of the global cancer burden [1]; however, research on hydrogel-based anticancer therapies is not evenly distributed across all cancer types. Many studies in the literature focus on the application of hydrogels for breast, colorectal, and lung cancers [26,27,28]. The high incidence of these types of cancers, as well as the therapeutic advantages of hydrogel systems, including local drug delivery, controlled release, and prolonged retention at the tumor site, contribute to this situation [8,9,10]. Hydrogel-based therapies are very attractive for neoplasms that face major clinical challenges, such as recurrence after surgery, systemic toxicity, and resistance to treatment [9,10]. Conversely, the limited evidence on the feasibility and clinical efficacy of localized biomaterial-supported treatment strategies for many cancers has led to relatively few studies targeting these cancers in hydrogel literature.

### 2.2. Current Treatments and Their Limitations

In cancer treatment, surgery, chemotherapy, radiation therapy, targeted therapies, and immunotherapy are widely used to halt or control the progression of the disease in many types of cancer [29]. Surgical treatment is one of the primary approaches for localized solid tumors; however, the risk of recurrence due to incomplete tumor resection poses significant limitations [30,31]. Chemotherapy can also affect healthy cells that divide rapidly, leading to various side effects [3]. In addition, cancer cells can expel the drug from the cell over time or develop resistance to chemotherapy [3,32,33]. Similarly, while radiation therapy works by causing DNA damage in tumor cells, it can also affect the surrounding healthy tissues [34]. Depending on the treatment site, skin reactions, gastrointestinal damage, and organ toxicity may occur [35]. Targeted therapies, however, act more selectively by inhibiting specific molecular targets and cause less damage to healthy cells [3,36]. However, the fact that a suitable molecular target is not present in every patient and that tumor cells can develop resistance to treatment over time are among the main challenges of this approach [33,36]. Similarly, while immunotherapy can produce long-term responses in some patients, it does not in all. Resistance and immune-related side effects are among the limitations of this treatment method [37,38].

All of these challenges encountered in current cancer treatments stem from factors such as the dynamic properties of the tumor microenvironment, metastatic potential, and treatment resistance—which hinder the effectiveness of current therapies [2,39]—as well as the inability of drugs to reach tumor tissue in sufficient quantities and their potential to cause unwanted toxic effects in healthy tissues [5,40]. This situation has become significant for the development of delivery systems and plant-derived bioactive compounds that can exert a more selective effect in cancer treatment, be applied directly to the target area, and release the drug in a controlled manner [4,5,9,10,11,12].

## 3. Plant Extracts and Plant-Derived Bioactive Anticancer Compounds

**Figure 2:**
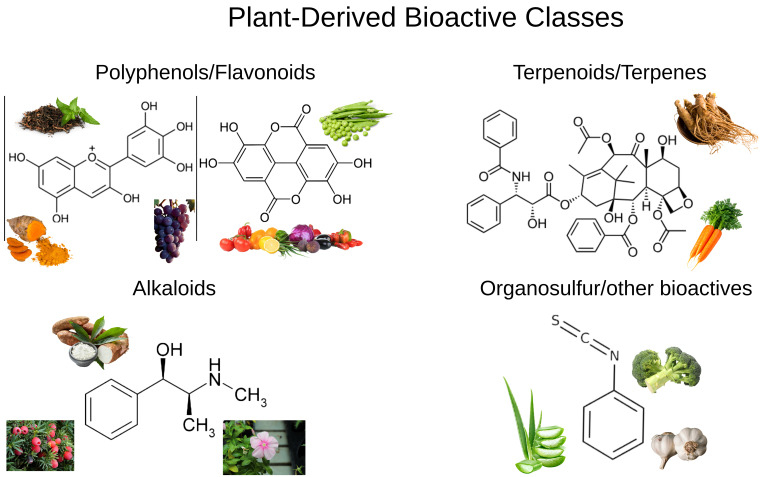
Classification of major plant-derived bioactive compounds with reported anticancer potential, including polyphenols, flavonoids, alkaloids, and terpenoid/organosulfur compounds.

Plant extracts, which serve as a natural source of compounds in cancer research, comprise various classes of secondary metabolites [41,42]. In this review, the terminology for plant-based therapeutic agents is used as follows. “Plant extracts” refer to complex mixtures obtained from plant materials that contain multiple chemical constituents. “Phytochemicals” are naturally occurring chemical compounds produced by plants, including polyphenols, flavonoids, alkaloids, terpenoids, and organosulfur compounds. The term “bioactive compounds” refers to phytochemicals that have demonstrated biological activity relevant to cancer therapy. “Plant-derived components” is used as a broader term encompassing both crude plant extracts and isolated plant-derived bioactive compounds. Consequently, plant extracts and plant-derived bioactive compounds are considered important in cancer research both as potential therapeutic agents and as adjunctive components that may support existing cancer treatments [43,44]. Figure 2 presents the classification of major bioactive groups under four main categories.

### 3.1. General Characteristics of Plant Extracts

Plant extracts are complex natural mixtures derived from various plant parts and can contain a wide range of phytochemicals, including biologically active compounds [42,45]. However, environmental conditions during the plant’s growth cycle, harvest, drying, and extraction influence the chemical composition of the plant extract [46,47]. In addition, the biological effects of plant extracts vary depending on the type and amount of the active compounds they contain, as well as their interactions with one another. This is important for the chemical characterization and standardization of plant extracts in pharmacological studies [45].

### 3.2. Major Phytochemicals with Anticancer Activity

Phytochemicals—compounds found in plants that can exhibit biological activity—include, in cancer research, primarily curcumin, resveratrol, quercetin, epigallocatechin gallate, genistein, luteolin, capsaicin, sulforaphane, berberine, mangiferin, vincristine, vinblastine, and paclitaxel [41,43,44,48,49,50]. The polyphenols and flavonoids in this group—including epigallocatechin gallate found in green tea, resveratrol found in grape skins, and quercetin found in various fruits and vegetables, and mangiferin, a C-glucosyl xanthone mainly isolated from *Mangifera indica* L.—are among the primary phytochemicals and are known as natural agents that have emerged as key players in anticancer research [51,52]. Mangiferin has attracted increasing attention because of its reported antioxidant, anti-inflammatory, anticancer, and chemopreventive activities [53,54,55]. Its anticancer relevance has been associated with the modulation of oxidative stress, inflammatory signaling, apoptosis, cell-cycle regulation, angiogenesis, and metastasis-related pathways [52,56]. While terpenoids and carotenoids are found in plants as pigments, flavor compounds, or defense molecules, alkaloids are nitrogen-containing, biologically active compounds, and organosulfur compounds are known as sulfur-containing natural compounds found particularly in garlic, onions, and certain allium species [42,57,58] (Table 1). Table 1 summarizes the findings in the literature regarding the association of bioactive groups with specific plant species and their proposed applications.

### 3.3. Mechanisms of Anticancer Action

Plant extracts and plant-derived bioactive compounds can act on cancer cells through various mechanisms [41,43,44]. The primary mechanism involves suppressing cell proliferation. This process is associated with a tendency to halt cancer cells in the G0/G1, S/M, or G2/M phases and slow their proliferation by affecting proteins and signaling pathways involved in the cell cycle [41,43].

**Figure 3:**
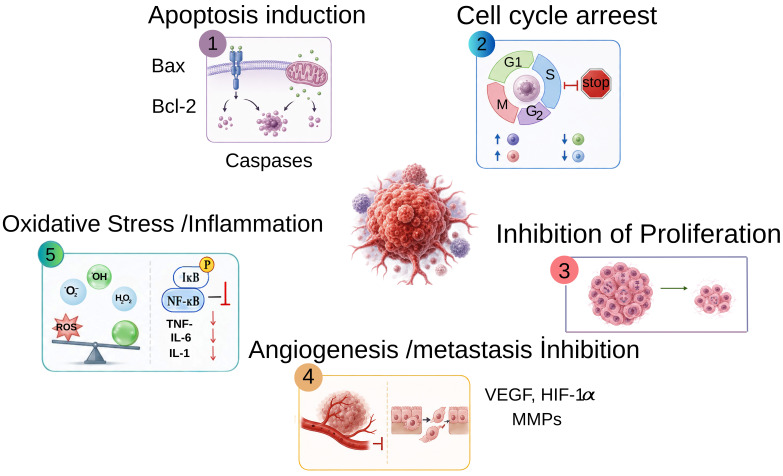
Mechanisms of action of bioactive compounds in cancer.

Another important mechanism is the induction of apoptosis [43,48]. This physiological process involves the programmed elimination of damaged or dysfunctional cells through bioactive compound-mediated activation of caspases in cancer cells, thereby affecting mitochondrial membrane potential or the balance between pro-apoptotic and anti-apoptotic proteins. Another mechanism of action is the regulation of oxidative stress [41,43,49]. This effect occurs because phytochemicals can either protect cells from oxidative damage by exerting antioxidant activity or trigger cell death by increasing reactive oxygen species (ROS) levels. Figure 3 illustrates the mechanism of action of bioactive compound-loaded structures in cancer.

Another anticancer mechanism, the suppression of angiogenesis, is reported to reduce the tumor’s ability to form blood vessels by affecting the activity of angiogenesis-related molecules, thereby limiting the supply of oxygen and nutrients to the tumor tissue. It has been reported that this mechanism may also reduce the migratory and invasive capacity of cancer cells through signaling pathways that suppress invasion and metastasis [41,43,44].

Finally, plant extracts and plant-derived bioactive compounds can also influence the tumor microenvironment through various interconnected mechanisms. These compounds can modulate oxidative stress and inflammatory signaling, regulate cytokine-mediated pathways, suppress angiogenesis-related factors such as VEGF, and influence processes associated with invasion and metastasis, including matrix remodeling and the epithelial–mesenchymal transition. In addition, some phytochemicals may modulate hypoxia-related signaling, cancer-associated fibroblast activity, and interactions among immune cells in the tumor microenvironment [2,39,41,43,44,48,49,50]. Therefore, the regulation of the tumor microenvironment should be interpreted not as a single effect, but rather as a multi-targeted process involving oxidative stress, inflammation, angiogenesis, extracellular matrix remodeling, hypoxia, and immune modulation.

### 3.4. Limitations of Plant Extracts

Although plant extracts and plant-derived bioactive compounds possess significant anticancer potential, their direct therapeutic use is subject to significant limitations [5,6]. These limitations stem from factors such as low solubility in water, low bioavailability, instability in the physiological environment, and the inability to reach the target tissue in sufficient quantities [6]. This situation reduces bioavailability by making it difficult for the active ingredient to reach the tumor tissue in sufficient quantities.

In addition, the complex structure of their extracts, variations in chemical composition, and the difficulty of standardizing dosages can pose major obstacles to reproducibility [45,46,47].

For this reason, the amount of active ingredients is important in chemical characterization. Furthermore, plant extracts need to be carefully evaluated for dosage, toxicity, and drug interactions. Some high doses of phytochemicals may have toxic effects or interact with chemotherapeutic drugs [61]. Therefore, the fact that these compounds are derived from natural sources does not necessarily mean they are always safe.

These limitations emphasize the necessity of delivery systems—such as nanocarriers, polymeric systems, and hydrogels—that can provide controlled release of plant extracts [5,6,9,10,62,63].

## 4. Properties of Hydrogel Systems and Their Roles in Cancer Treatment

**Figure 4:**
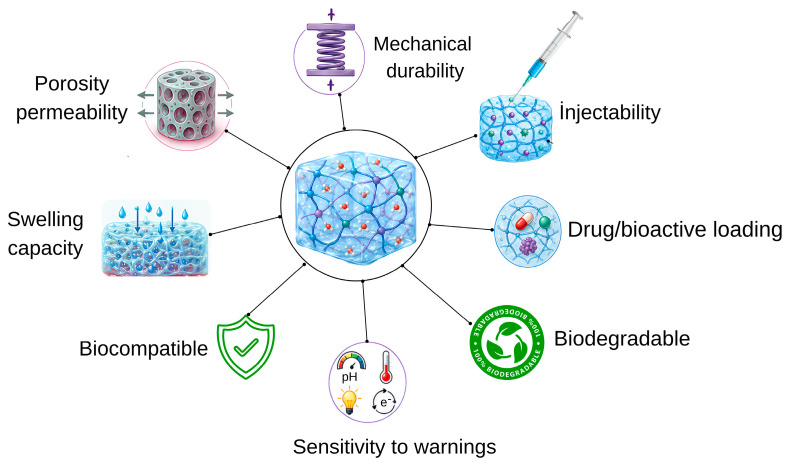
Properties of the hydrogel structure.

Hydrogels are three-dimensionally cross-linked polymeric networks that can retain large amounts of water and are also used as biomaterials [7,8,64]. The properties of hydrogel structures are shown in Figure 4. Hydrogel networks are formed through the physical or chemical cross-linking of polymer chains containing functional groups [7,8,64,65]. The structural and functional properties of hydrogels vary depending on the polymer source used, the chemical structure of the monomer, and the intended purpose of the hydrogel [7,64]. To this end, the polymer source, structural properties, cross-linking type, electrical charge, size, and stimulus sensitivity of hydrogel structures are of immense importance. These criteria are explained below under separate headings [64,65].

### 4.1. Types of Hydrogels

According to the sources, hydrogels can be divided into three groups: natural, synthetic, and hybrid [7,64]. Natural hydrogels are prepared from biopolymers such as alginate, chitosan, gelatin, hyaluronic acid, cellulose, dextran, and collagen. These hydrogels generally offer advantages in terms of biocompatibility and biodegradability. Synthetic hydrogels, on the other hand, are prepared from polymers such as PEG, PVA, polyacrylamide, polyacrylic acid, and PNIPAAm [7,8,64]. Synthetic hydrogels allow better control of mechanical properties, pore structure, and release rate. Hybrid hydrogels, on the other hand, are prepared by combining natural and synthetic polymers and offer advantages in terms of synergistic effects. Based on the classification approach, one may consider peptide-based hydrogels as a special type of supramolecular hydrogel systems. Peptide-based hydrogels are generally formed due to the self-organization of short peptides, peptide amphiphiles, or designed peptide molecules into three-dimensional hydrating networks. Such networks are stabilized via physical crosslinking owing to non-covalent interactions, including hydrogen bonding, electrostatic interactions, and π–π stacking interactions [66]. The result of such a self-assembly process is the capability of peptide-based hydrogels to possess high water content, injectability, biocompatibility, tunable mechanical stability, and mimicry of the extracellular matrix [67]. Moreover, the biodegradability, biological activity, possibility of synthetic preparation, and stimuli-responsive properties make such systems proper supramolecular structures for biomedical and drug delivery purposes [68]. Hence, peptide-based hydrogels may be seen as complementary materials to traditional natural, synthetic, and hybrid polymeric hydrogels within the general classification of hydrogels.

Based on their structural characteristics, hydrogels are classified into homopolymeric, copolymeric, multi-polymer network, and interpenetrating polymer network systems. While homopolymer hydrogels consist of a single type of monomer or polymer, copolymer hydrogels are formed by the copolymerization of two or more monomers [64]. In interpenetrating polymer network hydrogels, at least two different polymer networks are intertwined. Based on their size, hydrogels are classified as macrogels, microgels, and nanogels. While macro-gels are typically used as implants, wound dressings, tissue scaffolds, or local drug reservoirs, micro-gels and nano-gels are particularly important for drug delivery, cellular uptake, and application to the tumor microenvironment due to their small size [64]. Based on their electrical charge properties, hydrogels are classified as anionic, cationic, neutral, or amphoteric. While anionic hydrogels contain negatively charged functional groups, cationic hydrogels contain positively charged functional groups. This alters the electrostatic interactions between the hydrogel and the drug molecule or plant-derived active ingredient, as well as its loading capacity and release profile.

Therefore, the type of hydrogel should be selected based on the chemical structure of the active compound to be transported [8,64].

### 4.2. Types of Cross-Linking

In the literature, hydrogels are generally classified as physically, chemically, and dynamically cross-linked systems [64,65]. Physically cross-linked hydrogels are formed through mechanisms such as ionic interactions, hydrogen bonds, hydrophobic interactions, crystalline regions, chain entanglements, and temperature-dependent micelle formation among polymer chains [8,65]. However, hydrogels lack mechanical strength and long-term stability. Chemically cross-linked hydrogels are structures formed by the creation of covalent bonds between polymer chains. These systems offer advantages in terms of higher mechanical strength and long-term stability [8,65]. Dynamic cross-linked hydrogels are structures that can break down and reform in response to environmental conditions [69]. Examples of these types of bonds include disulfide bonds, boronate ester bonds, hydrazone bonds, and Diels–Alder reactions. These structures can exhibit self-healing, shape-changing, injectability, and stimulus-responsive release properties.

### 4.3. Stimulus-Responsive Hydrogels

**Figure 5:**
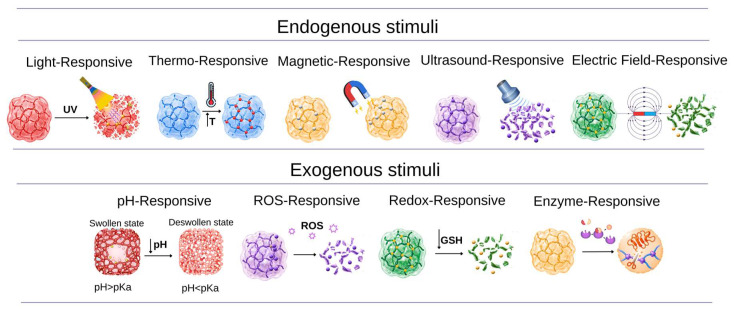
Comparative illustration of the response mechanisms of different stimulus-responsive hydrogel systems.

Structures capable of altering their degree of swelling, pore structure, mechanical properties, degradation rate, conductivity, or drug-release behavior in response to environmental or external stimuli are referred to as stimulus-responsive hydrogel systems [11,12,70]. These stimuli are generally classified into two groups: endogenous and exogenous stimuli. Endogenous signals include pH, redox potential, reactive oxygen species, enzymes, glucose levels, and biochemical changes specific to the tumor microenvironment. Exogenous stimuli, on the other hand, are external triggers, such as temperature, light, magnetic fields, electric fields, and ultrasound. Figure 5 illustrates the structural changes exhibited by hydrogel systems sensitive to pH, temperature, ROS, redox, enzymes, light, magnetic fields, ultrasound, and electric fields in response to specific internal or external stimuli.

pH-responsive hydrogels are one of the most commonly used smart hydrogel structures in cancer treatment. These are structures capable of responding to changes in the surrounding pH due to the presence of ionizable carboxyl, amine, phosphate, or sulfate groups in their polymer chains [11,70]. Temperature-responsive hydrogels are materials that exhibit a sol–gel or volume-phase transition in response to temperature. This property offers advantages in injectable formulations used in cancer treatment [71]. This hydrogel can be applied directly to the tumor site, where it then transforms into a gel to release the therapeutic agent. Redox-responsive hydrogels are particularly important for therapeutic agents, nucleic acids, or combination therapies that need to be delivered into cells. This approach is particularly important for intracellular drug release and the controlled delivery of chemotherapeutic agents [11,12,70].

It can also be used to enhance the intracellular release of plant-derived anticancer compounds [5,6,70]. Enzyme-responsive hydrogels are designed to respond to increased levels of specific enzymes in tumor tissue. By incorporating enzyme-degradable peptide or polysaccharide structures into the polymer network, it is possible to ensure that the hydrogel degrades only in the target area and enables controlled drug release. This approach offers an advantage in selectivity because it is designed to ensure release occurs only within the tumor region or the tumor-associated microenvironment [10,11,12,70]. ROS-responsive hydrogels are systems that respond to reactive oxygen species, which can increase in the tumor microenvironment or in inflammatory regions. In particular, the benefits of plant extracts—driven by certain natural compounds that regulate oxidative stress—offer a significant advantage in achieving synergy. Light-sensitive hydrogels are systems that react to light at a certain wavelength.

In these systems, photodegradable bonds, photothermal agents, or photodynamic components are utilized. Hydrogels containing photothermal agents damage tumor cells by inducing a local temperature increase upon light exposure and by enhancing drug release from the hydrogel [10,11,70]. Hydrogels sensitive to magnetic fields, ultrasound, and electric fields are among the systems that respond to external stimuli. Ultrasonic-sensitive systems, on the other hand, are devices that can be activated by transmitting sound waves from an external source [11,70].

### 4.4. The Functional Roles of Hydrogel Systems in Cancer Treatment

It is known that hydrogels, when used as delivery systems in cancer treatment, enable anticancer agents to remain at the tumor site for longer and be released in a controlled manner [9,10,70]. This enhances therapeutic efficacy and helps reduce toxic effects on healthy tissues. Another key feature is that they can be designed to be sensitive to their microenvironment. Thus, the active ingredient in the hydrogel will exhibit more pronounced release in the tumor region [10,11,12,70]. This holds significant potential, particularly for smart drug delivery systems. However, the ability of hydrogels containing plant extracts to maintain their structure within the matrix offers an advantage for more effective evaluation of their anticancer potential by facilitating their retention in the local area and controlled release [5,6,9,10,62,63]. It thus offers a solution-oriented approach to issues such as low solubility, low bioavailability, and stability [5,6,62,63].

## 5. Design and Evaluation Criteria for Hydrogels Loaded with Plant Extracts and Plant-Derived Bioactive Compounds

**Figure 6:**
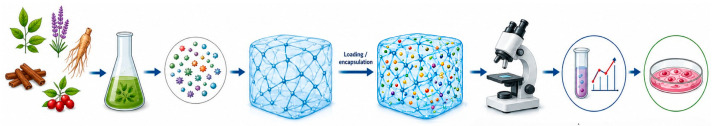
Schematic representation of the stages involved in the development of plant extract-loaded hydrogel systems, including extract selection, hydrogel matrix selection, loading/encapsulation, characterization, release analysis, and biological validation.

In the design of hydrogel systems, the incorporation of plant extracts or plant-derived bioactive compounds is not sufficient on its own [72,73]. For an effective design, the compatibility of the plant extract with the hydrogel structure must be evaluated alongside the extract’s chemical composition, physicochemical stability, release profile, and preservation of its therapeutic function [45,72,73]. Figure 6 illustrates the process leading up to the evaluation stage of plant extract-loaded hydrogel structures.

As a result, hydrogel functions as an active functional platform rather than merely a passive carrier. In conclusion, the question “Which extract should be combined with which hydrogel system, and why?” must be addressed holistically [72,73].

### 5.1. Factors Influencing the Selection of Plant Extracts/Bioactive Compounds

The first step in hydrogel design is selecting plant extracts. The fact that an extract is of natural origin alone is not sufficient justification for its use in hydrogel formulations [61,74]. The extract’s anticancer potential should be evaluated based on key parameters, including the effect of its active ingredient on cancer cells, its safety margin, and its suitability for formulation. In general, when selecting an extract, the decision should be based on the intended biological effect, the extract’s phytochemical composition and standardization, its solubility and stability properties, and its safety and selectivity profile [45,61,74]. In this context, standardization should not be limited to the general use of “standardized extracts” but should be considered a multi-level requirement in plant-extract selection and hydrogel formulation design [75,76,77]. Future studies should clearly report botanical authentication of the plant source, the plant part used, extraction solvent and conditions, extract yield, phytochemical fingerprinting, marker compound quantification, and batch-to-batch reproducibility [75,76,77]. In addition, the chemical profile of the extract should be correlated with its biological activity whenever possible, and this relationship should be re-evaluated after incorporation into the hydrogel matrix [64,66,67]. For hydrogel-based systems, standardization should also include loading efficiency, release kinetics, swelling/degradation behavior, physicochemical stability, and protection of encapsulated bioactive constituents until release [73,78]. This approach would improve reproducibility, enable comparisons between studies, and strengthen the reliability of plant extract-loaded hydrogel systems [75,76,77,78].

### 5.2. Compatibility of the Extract or Bioactive Compound with the Hydrogel Matrix

The compatibility of the extract and hydrogel must be evaluated in conjunction with the chemical properties of the selected polymer matrix, the solubility of the extract components, and their molecular interactions. As a result, compatibility directly affects loading efficiency and release behavior, which supports therapeutic efficacy [72,73].

In particular, when evaluating hydrogel–extract compatibility, one should not rely solely on the question “Can they be mixed?” but rather proceed based on the answer to the question “Does the active ingredient remain functional in the formulation?” [45,72,73].

### 5.3. Parameters Indicating Formulation Success

For a hydrogel containing plant extracts or plant-derived bioactive compounds to be considered successful, a consistent relationship must be established between its physicochemical performance and biological activity. The primary factors influencing this situation are loading capacity and loading efficiency [73]. While loading capacity indicates the amount of active ingredients or extract that a hydrogel can carry per unit mass, loading efficiency refers to the ability of a hydrogel to absorb extract. In addition, the hydrogel’s swelling ratio is also an important parameter because it directly affects the movement of the active ingredient within the hydrogel and its release into the external environment [72,73]. Other important factors—such as the hydrogel’s degradation or erosion behavior, mechanical and rheological properties, morphological and structural characterization, release profile and kinetics, and biocompatibility—must be evaluated collectively to assess formulation success.

### 5.4. Therapeutic Interpretation of Release Data

In hydrogels containing plant extracts or plant-derived bioactive compounds, two phases are most commonly observed during release. In the first phase, components that are near the surface or weakly bound are rapidly released. This is called a burst release [72,73]. In the second phase, the components retained in the inner regions of the hydrogel diffuse more slowly, resulting in a sustained-release profile [72,79]. A rapid initial dose may be advantageous in reaching the therapeutic threshold. It should be evaluated appropriately in terms of dosage and intended use. However, the excessive burst-release toxicity and short duration of action indicate that the formulation has failed. Other influencing factors include the biological characterization of the release medium, the mechanistic interpretation of release kinetics, and the ability of the released extract to maintain its biological activity [73,79,80].

In conclusion, the release of plant extracts from hydrogels should not be analyzed solely by the question “What percentage was released over how many hours?” Rather, release data should be evaluated alongside factors such as therapeutic duration, effective dose range, toxicity threshold, target tissue, and biological activity.

### 5.5. Correlation Between Biological Activity and Hydrogel Performance

In hydrogel systems containing plant extracts or plant-derived bioactive compounds, biological activity should be evaluated alongside hydrogel performance. First, cytotoxicity and the release profile should be addressed [73]. In addition, control groups should be designed to determine whether biological activity is due to the extract alone or to the hydrogel–extract combination. In studies, the free extract, blank hydrogel, extract-loaded hydrogel, and, if available, a commercial comparator product should generally be evaluated together [73]. In addition, it is important to establish a dose–response relationship. In vivo data should be evaluated with consideration of differences between 2D cell culture results and 3D models [81,82].

## 6. The Use of Hydrogels Containing Plant Extracts and Bioactive Compounds in Cancer Treatment

Various studies demonstrate the potential of hydrogel systems containing plant extracts and plant-derived bioactive compounds for cancer treatment. In this context, hydrogel systems containing plant extracts and plant-derived bioactive compounds obtained from turmeric, grape/grape skin, green tea, aloe vera, onion, and citrus and mango/*Mangifera indica* L. sources, as reported in the literature, have been comprehensively investigated [62,63,83,84,85,86,87,88,89,90,91,92,93,94,95,96,97,98,99,100,101,102]. Table 2 presents the design of hydrogels incorporating bioactive agents and their applications in cancer treatment.

Rather than interpreting these studies as independent examples, the hydrogel systems summarized in Table 2 should be evaluated according to three comparative criteria: the type of plant-derived bioactive compound, the functional role of the hydrogel platform, and the level of therapeutic evidence. From this perspective, curcumin-based studies mainly illustrate the use of hydrogels to overcome poor solubility and bioavailability; resveratrol, EGCG, quercetin, and naringenin systems emphasize controlled release, local retention, or stimulus-responsive delivery, while Aloe vera- and extract-based hydrogels often function as biocompatible matrices, supportive platforms, or tumor-modeling systems rather than fully validated therapeutic systems [83,84,85,86,87,88,89,90,91,92,93,94,95,96,97,98,99,100]. Therefore, the following studies are discussed not only as individual formulations but also in terms of what each platform contributes to cancer-related delivery, where its advantages lie, and what limitations remain.

Curcumin, the primary bioactive component of turmeric, is frequently used in cancer treatment research due to its antiproliferative, proapoptotic, antioxidant, anti-inflammatory, and anti-angiogenic effects [13,48,83,86]. Curcumin has emerged as a leading candidate in hydrogel-based cancer research; in these studies, it serves both as a bioactive anticancer phytochemical and as a well-defined model compound for formulation development. Unlike crude plant extracts, which vary depending on botanical origin, cultivation conditions, and harvesting, processing, and extraction methods [45], curcumin is a chemically well-defined compound that can be consistently loaded, released, and evaluated. Furthermore, its hydrophobicity, low water solubility, instability, and poor bioavailability make it a suitable model for evaluating the potential of hydrogel matrices to improve the delivery of plant-derived bioactive compounds that are poorly soluble in water through localized retention, protection, and sustained release [13]. Curcumin is mechanistically involved in cancer-related processes such as proliferation, apoptosis, oxidative stress, inflammation, and angiogenesis; this evidence justifies its use in various cancer models [48]. Consequently, the reason for curcumin’s widespread presence in the literature is based not so much on its superiority over other plant-derived bioactive compounds in a clinical setting but rather on its chemical definability, compatibility with formulations, and comprehensive mechanisms of action [13,48,83,84,85,86].

One of the matrices most commonly used for this purpose is the alginate–chitosan hydrogel system. Abbasalizadeh and colleagues prepared hydrogels using the CaCl_2_-mediated ionic gelation method to investigate their effects on the breast cancer cell line T47D and the lung cancer cell line A549; they performed MTT, DAPI staining, and cell cycle analyses. The results showed that curcumin/chrysin-loaded alginate–chitosan hydrogels reduced cancer cell viability, induced apoptosis, and caused cell-cycle arrest in the G2/M phase in A549 and T47D cells, thereby demonstrating a direct anticancer effect in cancer cell lines [83]. In another study, an alginate hydrogel matrix was prepared by loading curcumin onto graphene oxide nanolayers and then cross-linking it with calcium ions to enable local treatment of squamous cell carcinoma lesions. It has been demonstrated that graphene oxide, incorporated into the matrix structure, enhances hydrogel stability, while curcumin may be suitable for local/topical cancer treatments by inducing cytotoxic effects in cancer cells [84]. In a study aimed at exploiting biochemical differences in the tumor microenvironment, a supramolecular hydrogel system containing curcumin modified with glycyrrhetinic acid was developed and reported to inhibit the proliferation of HepG2 liver cancer cells [85]. George and colleagues developed a chitosan/ZnO-based nanocomposite hydrogel cross-linked with biomass-derived dialdehyde cellulose for the delivery of curcumin. The curcumin-loaded hydrogel enhanced the biological activity of curcumin compared to free curcumin and exhibited a stronger cytotoxic effect against A431 human skin carcinoma cells. These findings suggest that this system holds potential as a hydrogel-based platform for the delivery of plant-derived anticancer compounds [86]. Among the conventional polymeric and nanocomposite hydrogel matrices used for curcumin delivery, alginate–chitosan hydrogel systems are among the most widely researched platforms. While curcumin-loaded hydrogel delivery systems are primarily prepared using polymer and/or nanocomposite materials, peptide-based hydrogels have been investigated as supermolecular formulations for the hydrophobic biologically active compound derived from these plants. For example, Chen et al. [103] characterized the development and microstructural formation, mechanical properties, and curcumin encapsulation ability of a pH-sensitive peptide hydrogel. It is important for hydrogel-based delivery because it demonstrates that a hydrophobic phytochemical can be incorporated into a peptide-based hydrogel system [103]. In this regard, peptide-based hydrogels can be considered alternative platforms that expand the existing formulation strategies for curcumin due to their supramolecular structures.

Grape skin and seed are rich sources of bioactive polyphenols, including resveratrol and oligomeric proanthocyanidins, which have attracted attention in cancer research due to their antioxidant, anti-inflammatory, and anticancer effects [48,87,88,89]. A review of the literature reveals that only a limited number of studies have used grape extract directly. However, hydrogel systems loaded with resveratrol—an active component of grapes—are among the most widely studied [87,88]. To this end, Kotta and colleagues designed a thermosensitive hydrogel system loaded with resveratrol nanoemulsion for breast cancer. Analyses were conducted on the MCF-7 breast cancer cell line, and the system was observed to exert a cytotoxic effect on cancer cells [87]. In a similar study, Shin and colleagues developed click-crosslinked hyaluronic acid hydrogel structures containing resveratrol for intratumoral administration in a mouse model of breast cancer. The results indicate that the resveratrol-loaded hydrogel remains in the tumor site for a longer period, increases the number of apoptotic cells, and inhibits angiogenesis, thereby suppressing tumor growth [88]. In another study, a hydrogel scaffold structure inspired by grape seeds—which exhibit photothermal properties—and containing OPC was designed for the treatment of melanoma and wound healing. This study is significant in that hydrogels containing plant polyphenols can serve not only as passive drug carriers but also as a photothermal therapy platform [89]. Green tea (*Camellia sinensis*) extract and its major polyphenolic constituent, epigallocatechin gallate, have attracted attention in cancer research due to their antioxidant, anti-inflammatory, antiproliferative, anti-angiogenic, and pro-apoptotic effects [49,90,91]. To take advantage of these biological effects, Yu and colleagues developed a gellan gum/chitosan-based bilayer scaffold containing green tea and curcumin. Analysis of the MCF-7 breast cancer cell line revealed decreased cell viability and increased antioxidant and antibacterial activity. While the release profile for green tea was short-lived, that of curcumin was more sustained [90]. In another study, a structure containing EGCG, indocyanine green, and gold nanoparticles was designed to develop hydrogel systems sensitive to the tumor microenvironment in breast cancer. While the indocyanine green contained in the matrix exerts photodynamic/photothermal effects, EGCG has been reported to exhibit protein-inhibiting and anticancer properties. This study demonstrates that smart hydrogel systems containing EGCG can be used in multimodal cancer therapy due to their ability to reduce tumor size and to prevent recurrence [91].

In recent years, Aloe vera gel and extracts—also known as adjuvants or biological modulators in cancer treatment—have been found to be complex structures containing polysaccharides, particularly acemannan, anthraquinone derivatives, phenolic compounds, vitamins, and amino acids [92,93]. To harness this biological potential of aloe vera, Charron and colleagues developed aloe–alginate hydrogels for the treatment of cervical cancer. The findings indicate that hydrogel can reduce cervical cancer cell viability and that aloe vera may act as a bioactive component rather than merely serving as a supporting matrix [92]. In a separate study, Preda and colleagues investigated the potential for tumor spheroid formation using MDA-MB-231 breast cancer and U87MG glioblastoma cells by designing hydrogels based on natural components such as alginate, aloe vera gel powder, and chitosan—primarily for tumor modelling or as a 3D tumor spheroid culture platform rather than for cancer treatment. In conclusion, this can also serve as an in vitro platform for modeling the tumor microenvironment [93]. These findings indicate that Aloe vera-containing hydrogels may function not only as bioactive systems but also as biocompatible matrices, supportive carriers, or tumor-modeling platforms [81,82]. Within this context, flavonoid-loaded hydrogel systems, particularly those based on quercetin, have mainly been investigated as strategies to improve solubility, biocompatibility, and controlled release while preserving anticancer activity [94,95,96]. Onion skins, a plant-based waste source, are rich in quercetin; therefore, quercetin is frequently evaluated as an onion-derived flavonoid in hydrogel-based anticancer formulations [94].

George and colleagues developed a quercetin-loaded chitosan–cellulose/ZnO nanohybrid hydrogel for the treatment of skin cancer. According to the analysis, the hydrogel system exhibited biocompatibility in L929 fibroblast cells and anticancer activity against A431 human skin carcinoma cells [94]. In another similar study, Kundrapu and colleagues developed a pH-sensitive injectable hydrogel containing quercetin and taxifolin for the treatment of breast cancer. The analysis results were evaluated using the triple-negative breast cancer cell lines MDA-MB-231 and MDA-MB-468. The results showed that it enables controlled release at low pH and induces cytotoxicity, cell-cycle arrest, apoptosis, ROS production, and reduced migration and mammosphere formation in TNBC cells [95]. In another study, a quercetin-loaded xanthan gum/guar gum/halloysite nanotube hydrogel system was developed for liver cancer cells. The results showed pH-sensitive release and a selective apoptotic effect on HepG2 liver cancer cells [96]. Veetil and colleagues prepared sodium alginate/gelatin hydrogel beads incorporating biogenic silver nanoparticles synthesized using Clitoria ternatea plant extract. The developed system exhibited anticancer activity against A549 lung cancer cells; MTT and AO/EB staining results revealed dose-dependent cytotoxicity and apoptosis-related cell death. These findings support the potential of this structure as a plant extract-mediated hydrogel bead platform for cancer-related applications [97].

Due to its low solubility and limited bioavailability, naringenin, another bioactive compound, has been delivered via hydrogel and nanohydrogel systems [98,99,100].

To this end, George and colleagues developed a chitosan-based nanohybrid hydrogel containing zinc oxide nanoparticles to treat skin carcinoma cells. The study demonstrated that hydrogel/nanohybrid carriers can enhance the therapeutic effect of low-solubility citrus flavonoids [98]. In a similar study, Sharma and colleagues developed Pluronic F127 hydrogel systems containing naringenin nanocrystals for the treatment of skin cancer. The results demonstrated that the Pluronic F127 hydrogel system constitutes an improved topical delivery platform in terms of solubility, release, and skin permeability and that it exhibits anticancer activity in skin cancer cells through mechanisms involving oxidative stress and apoptosis [99]. In another study, Md and colleagues designed a pH-responsive bilayer nano-hydrogel structure based on a naringenin/protein-polysaccharide complex targeting colorectal cancer cells. The results obtained indicate that naringenin increases solubility, exhibits cytotoxicity, and enables pH-selective release [100].

Mangiferin is a bioactive substance of natural origin that belongs to the class of C-glucosyl xanthones and is mostly extracted from *Mangifera indica* L. It is a promising candidate for hydrogel carriers because of its anticancer activity, poor water solubility, and low oral bioavailability [51,56]. According to studies by Morozkina et al., mangiferin has anticancer activity but also has limitations, such as limited absorption and bioavailability; therefore, it is essential to evaluate it in combination with polymeric carrier systems [51]. In their work, Sarfraz et al. pointed out that solubility and bioavailability of mangiferin are the main difficulties of its formulation for cancer treatment, and the development of nanotechnology-based carrier systems is ongoing [56].

As for the application in hydrogels, the in situ hydrogel formulation was synthesized by Meng et al. using the self-assembling peptide RADA16-I in order to deliver hydrophobic mangiferin in aqueous media Meng et al. [101]. This study involved examinations of hydrogel formation and release behavior of mangiferin, and in vitro assessment of cell viability. As shown by the results, the use of RADA16-I mangiferin hydrogel enables regulation of mangiferin release, enhances suppression of tumor cell proliferation compared with free mangiferin, and reduces toxicity to normal cells [101]. In the same context, the phospholipid-based formulation of mangiferin nanohydrogel was developed by Alkholifi et al. to overcome oral bioavailability issues and enhance topical/local application. According to the authors’ findings, the structure exhibits Fickian release behavior, cellular absorption increases by nearly 3-fold, and in vitro anticancer activity increases by approximately 4-fold [102]. However, it has been emphasized that formulation studies focusing on solubility, bioavailability, and carrier system design should be further developed in order to translate the antitumor potential of mangiferin into clinical applications [56,104].

pH-responsive hydrogel was formulated using an extract of Parthenocissus quinquefolia L. as a natural cross-linker with antioxidant and biodegradable properties. The plant extract was incorporated as a bioactive agent into the formulation, thereby forming a hydrogel network structure. The results showed that the hydrogel exhibited controlled-release behavior and anticancer activity. These results suggest that plant extracts can serve as both structural and therapeutic components in hydrogel-based cancer drug delivery systems [62]. In another study, the Ozay group prepared a poly (acrylic acid-co-2-hydroxyethyl methacrylate)-based hydrogel with antioxidant and biodegradable properties, using rutin as a crosslinking agent. Rutin was used as a bioactive and structural component in the hydrogel matrix, providing the antioxidant feature to the system. The results showed that the rutin-crosslinked hydrogel had the capacity to release the drug and anticancer activity [63].

Studies have shown that hydrogel platforms differ not only in compositional properties but also in therapeutic problems that they are targeting. Natural polymer-based hydrogels, such as alginate, chitosan, gelatin, gellan gum, and other polysaccharide systems, possess major advantages such as biocompatibility, biodegradability, suitable formulation conditions, high water content, and suitability for localized drug release [7,64,65,72]. However, for systems with natural polymers or plant-derived constituents, the main drawbacks are the standardization of raw materials, variability in extract and phytochemical composition, lack of chemical characterization, and quality control requirements [45,46,47,75,76,77]. Additionally, these hydrogel systems may also encounter some formulation-related problems such as poor control of mechanical strength, swelling behavior, degradation rate, burst release, and drug release kinetics [8,64,72,73]. Thermosensitive and injectable hydrogels are advantageous systems, particularly for local cancer treatment, because they permit minimally invasive application, allow for in situ depot formation, and ensure localized and sustained drug release at the tumor site [71,87,88]. But the therapeutic efficiency of these systems relies on a number of parameters, for example, the efficiency of gelation, residence time at the application site, distribution within the tumor tissue, and penetration beyond the local application area. Thus, a successful in vitro release profile should not always be considered as an indicator of better in vivo antitumor efficacy [71,73]. Nanocomposite hydrogels with graphene oxide, ZnO, gold nanoparticles, silver nanoparticles, or halloysite nanotubes have been developed to enhance mechanical stability, drug loading capacity, controlled release, photothermal/photodynamic response, or multimodal anticancer effects [5,84,86,91,96,97]. However, the nanomaterial-based systems have significant translational challenges, including long-term biosafety, potential toxicity of nanomaterials, biodistribution, clearance, complexity of manufacturing, and clinical scalability [5,73]. pH-responsive hydrogels, in turn, can achieve selective release by making use of the acidic tumor microenvironment or pH variations in the gastrointestinal tract [11,12,70]. However, pH-dependent release is often evaluated under simplified in vitro conditions, and such release profiles can be interpreted using established kinetic models [79,80]. Therefore, these in vitro results alone cannot demonstrate in vivo selective release, tumor-specific accumulation, or superior antitumor efficacy [70,73].

In this regard, the main issue in plant-derived anticancer hydrogel systems is often not direct experimental failure but rather the insufficient demonstration of therapeutic superiority under biologically complex and clinically relevant conditions [73,81,82]. Nevertheless, some studies also indicate that certain formulation approaches may not provide the expected added benefit. For example, the addition of doxorubicin in an aloe–alginate hydrogel system did not produce a significant additional effect on cancer cell viability compared to the formulation containing only aloe, revealing that natural component-hydrogel combinations do not always yield synergistic results [92]. Although many experimental studies report improved formulation behavior, sustained release, or localized application advantages, direct comparisons with free compounds, non-hydrogel carrier systems, or standard treatment approaches remain limited [83,84,85,86,87,88,89,90,91,92,93,94,95,96,97,98,99,100].

Furthermore, most of the current evidence is on in vitro or early preclinical models. Moreover, the lack of evaluation of translationally critical parameters such as pharmacokinetics, biodistribution, tumor penetration, systemic toxicity, recurrence, and long-term safety [5,6,73,81,82] is also a concern. In conclusion, the present literature indicates the potential of hydrogel platforms for plant-derived anticancer agents. However, it has not yet been clearly established which hydrogel platform is the most suitable option for a specific compound, cancer type, route of administration, or therapeutic target [9,10,71,72,73].

## 7. Hydrogels Containing Plant Extracts, Plant-Derived Bioactive Compounds, and Anticancer Drugs and Their Synergistic Effects

The incorporation of plant-derived bioactive compounds into hydrogel systems, in combination with chemotherapeutic drugs, has garnered attention for its potential to enable more effective cancer treatment strategies. Table 3 summarizes the studies in the literature that address this objective. Hydrogels containing bioactive compounds and anticancer drugs have various applications in cancer, as outlined in Table 3.

The studies listed in Table 3 are discussed below in terms of their bioactive compound–drug combinations, hydrogel platforms, and reported therapeutic outcomes.

The studies summarized in Table 3 demonstrate the potential of combining plant-derived bioactive compounds with anticancer drugs in hydrogel systems. However, a direct comparison of loading capacities across these systems remains difficult because the studies use different polymer compositions, co-loading strategies, drug-to-bioactive compound ratios, and reporting units. Therefore, the available data do not allow a definitive ranking of hydrogel types according to loading capacity. Instead, the main distinction among these systems appears to be functional: some formulations are designed primarily to improve co-loading of poorly soluble phytochemicals with chemotherapeutic agents, whereas others aim to prolong local retention, control release, reduce systemic toxicity, or support tumor-associated delivery. Thus, formulation complexity should not be interpreted as direct evidence of higher loading capacity or superior therapeutic efficacy. Accordingly, the studies listed in Table 3 are discussed below in terms of their bioactive compound–drug combinations, hydrogel platforms, functional delivery purposes, and reported therapeutic outcomes.

In this context, imatinib-loaded Aloe vera/sodium alginate/PVA hydrogels have been developed. The study investigated pH-sensitive drug release from hydrogels, improvements in their biochemical properties, and their cytotoxicity against breast cancer cells. The results indicate that the aloe vera structure provides biological support to the hydrogel matrix and that the targeted anticancer activity is enhanced in the drug-loaded aloe vera/imatinib hydrogel [105]. In another study, an aloe vera/doxorubicin/hydrogel structure was developed to enable the controlled release of the anticancer drug. The designed structure consists of doxorubicin-loaded bacterial ghost carriers embedded in a natural hydrogel matrix. The study found that aloe vera provides a matrix with high water-holding capacity and has a regulatory effect on doxorubicin release [106]. Doxorubicin-loaded chitosan/graphene/cellulose nanowhisker hydrogel structures were developed in this study; doxorubicin and curcumin were co-administered to reduce resistance and side effects associated with single-drug therapies. It has been reported that, due to its pH-responsive hydrogel nature, it enables more controlled drug release under acidic conditions and that its antitumor effect is enhanced by the doxorubicin-curcumin combination [107]. In a study conducted for the same purpose, a doxorubicin-loaded chitosan hydrogel was developed to enable sustained local drug release and reduce systemic side effects, with both agents co-loaded onto the same hydrogel platform. It has been observed that, due to its pH-sensitive structure, drug release increases in the acidic tumor microenvironment, thereby reducing side effects [108]. In another study, a self-assembled peptide hydrogel structure was developed for the treatment of head and neck cancer, loaded with doxorubicin. Doxorubicin and curcumin have been co-loaded into the hydrogel matrix. The study found that a bifunctional peptide hydrogel can control the release rate of drugs based on their differences in water solubility and more effectively inhibit cell growth in head and neck cancer cells [109].

Following the self-assembling peptide hydrogel example for curcumin–doxorubicin co-delivery, Gallo et al. [110] further extended this strategy to a nanoscale Fmoc-FF peptide-based nanogel system in thyroid cancer cells. Within the scope of this research, the authors have synthesized Fmoc-FF hydrogel-derived nanogels and investigated the behavior of this system with the help of doxorubicin, curcumin, and fluorescently labeled compounds as models. The results revealed that Fmoc-FF nanogels can transport curcumin with high encapsulation efficiency, provide stable nanosized architecture, and offer a slow/sustained release profile for curcumin. Moreover, it has been reported that nanogels can be internalized by the thyroid cancer cells and delay nuclear uptake of doxorubicin in comparison with free doxorubicin. According to cell viability studies, doxorubicin-loaded nanogels exert cytotoxic effects on thyroid cancer cells similar to those of free doxorubicin, although curcumin-loaded nanogels can decrease the cell viability, and their activity is less than that of free curcumin [110].

Doxorubicin-loaded gelatin–oxidized alginate hydrogel was developed in this study with the aim of developing a local combination therapy for breast cancer and enhancing the drug’s efficacy with quercetin. Doxorubicin was loaded onto the hydrogel phase, while quercetin was loaded onto chitosan-coated zein nanoparticles. It has been reported that the study offers an advantage by ensuring the controlled release of the two agents and supporting a combined cytotoxic effect in breast cancer cells [111]. A doxorubicin-loaded, pectin-based hydrogel has been developed to treat lung tumors and provide synergistic therapy with the bioactive compound limonin. Doxorubicin is covalently bound to the hydrogel matrix, while limonin is loaded into the system. The authors reported a synergistic anticancer response for the doxorubicin–limonin hydrogel system, together with inhibition of lung tumor growth [112].

Doxorubicin-loaded chitosan/albumin/hydroxypropyl-β-cyclodextrin nanogel is designed to reduce side effects, including neurotoxicity. Resveratrol and the drug have been encapsulated together in the resulting formulation. The results have shown that the protective/antioxidant activity of doxorubicin and resveratrol, which exhibit pH-dependent release, may reduce side effects while providing therapeutic benefits [113]. Paclitaxel-loaded Fucoidan–Pluronic F127 nanogel was developed in a study conducted for the treatment of breast cancer. Paclitaxel and curcumin have been co-loaded onto the structure. The study found that the dual-loaded nanogel exhibited controlled release at acidic pH. It has also been noted that curcumin may provide a more balanced and sustainable treatment by modulating paclitaxel toxicity [114]. Paclitaxel-loaded heparin–Poloxamer P403 nanogel was developed in this study for the treatment of breast cancer. It was administered in combination with paclitaxel and curcumin. The study reported enhanced inhibition of breast cancer cells for the paclitaxel–curcumin nanogel system, which was attributed to a synergistic combination effect, together with pH-sensitive release behavior [115]. In the study in which the heparin–Poloxamer P403 nanogel structure was developed, platinum hydrate-loaded curcuminoids and cisplatin hydrate were co-loaded into the structure to reduce the side effects of platinum therapy and enhance its antitumor efficacy. The study suggested that the platinum–curcuminoid combination contributed to enhanced antitumor activity, controlled release, and reduced platinum-associated side effects [116]. Lauroyl-gemcitabine/GemC12-loaded curcumin/nanocapsule-hydrogel constructs are designed to target GSC-like cells responsible for tumor recurrence using the same local platform for the treatment of glioblastoma. It has been noted that the results obtained may hold promise for preventing glioblastoma recurrence [117]. This study developed an oxaliplatin-loaded hyaluronic acid-based hydrogel to eliminate residual tumor cells following colorectal cancer surgery and to prevent peritoneal metastasis. Oxaliplatin and curcumin have been incorporated into the hydrogel. The results obtained have led to the development of a hydrogel strategy for sustained pH-responsive release and for preventing postoperative recurrence and metastasis [118]. A 5-fluorouracil-loaded silk fibroin hydrogel was developed to enhance 5-fluorouracil efficacy and reduce its toxicity in the treatment of colorectal cancer. 5-fluorouracil and curcumin were co-loaded into the hydrogel system. The results indicated that curcumin enhances the anticancer effect of 5-fluorouracil, while silk fibroin hydrogel provides controlled release and results in lower toxicity due to its therapeutic selectivity [119]. For colorectal cancer therapy, a Schiff-base cross-linked injectable hydrogel was designed to co-deliver 5-fluorouracil and curcumin within the same delivery system. A micellar approach using Pluronic F127 was chosen for hydrophobic curcumin, while a hydrogel network structure was selected for the hydrophilic 5-fluorouracil compound. Analysis results showed that the 5-fluorouracil–curcumin co-delivery hydrogel system exhibited enhanced anticancer effects in HT-29 colorectal cancer cells, with confirmed synergistic inhibitory effects on cell-cycle progression and cell proliferation [120]. 5-fluorouracil-loaded gelatin-based hydrogels and microgels were developed in this study to enhance the efficacy of colorectal cancer treatment. A hybrid delivery system was created by co-loading the structure with 5-FU and curcumin. It has been reported that this system is suitable for local and long-term combination therapy [121]. In another study, doxorubicin-loaded poly(3-sulfopropyl acrylate)/pectin-based hydrogels were developed as a sustained drug delivery system for anticancer therapy [106]. The hydrogel matrix was formed using the plant polysaccharide pectin, which incorporated silver nanoparticles and quantum dots to enhance the system’s functionality. The results showed sustained release of doxorubicin, indicating the hydrogel can be a promising platform for prolonged local delivery of anticancer drugs [122].

Taken together, these studies indicate that combination hydrogel systems provide different types of therapeutic benefit, ranging from controlled release and prolonged local delivery to toxicity reduction and enhanced anticancer activity. Regarding therapeutic efficacy, evidence for synergistic activity should be interpreted according to the experimental controls and comparative data provided in each study. Stronger evidence is obtained when the combined hydrogel system is compared with the free drug, the free bioactive compound, single-agent-loaded hydrogels, and blank hydrogel controls [73]. In several studies, the reported benefit mainly involves improved release behavior, reduced toxicity, sustained local delivery, or supportive local treatment potential rather than quantitatively confirmed synergy [106,113,117,122]. Therefore, combinations such as curcumin–doxorubicin [107,108,109], curcumin–paclitaxel [98,99], quercetin–doxorubicin [111], and limonin–doxorubicin [112] should be interpreted according to the direct comparative data and the type of therapeutic outcome reported in each study, rather than being uniformly regarded as confirmed synergistic systems. Accordingly, within the reviewed studies, stronger evidence of reported synergistic or enhanced combined therapeutic efficacy is mainly reported for curcumin–doxorubicin self-assembling peptide hydrogels [109], curcumin–paclitaxel systems [114,115], limonin–doxorubicin hydrogels [112], curcuminoid–cisplatin nanogels [116], and curcumin–5-fluorouracil systems [120,121].

In contrast, aloe vera/doxorubicin systems, resveratrol/doxorubicin nanogels, curcumin/GemC12 nanocapsule-hydrogel constructs, and pectin-based doxorubicin systems more clearly demonstrate delivery-related benefits, sustained release, toxicity reduction, or supportive local delivery rather than uniformly confirmed quantitative synergy [106,113,117,122].

### Cancer-Type-Specific Interpretation of Hydrogel-Based Strategies

In previous sections, hydrogel systems were evaluated based on the plant-derived bioactive components loaded, combinations with anticancer drugs, and formulation approaches. In this section, the current literature’s positioning across cancer types is discussed. Such an evaluation is important for showing which tumor models hydrogel-based approaches have been investigated more intensively and for which cancer types the evidence remains more limited. Table 4 presents an integrated overview of the examined studies categorized by cancer type.

Overall, this distribution indicates that the relevance of plant-derived component-loaded and combination hydrogel systems depends not only on the hydrogel platform or loaded agent, but also on the cancer model and therapeutic context. Moreover, this cancer-type-specific framing provides a conceptual bridge to the limitations and future research directions addressed in Section 8.

## 8. Future Directions

In line with botanical drug development principles, utilizing standardized extracts in extract- and component-focused formulations within hydrogel systems containing plant extracts would represent a significant advancement. This approach may enhance comparability across studies and contribute to a more reliable interpretation of the formulation’s biological effect [123,124]. Another important parameter is the development of multi-stimulus-responsive hydrogels that are intelligent and suited to the complex structure of the tumor microenvironment [125,126,127]. This development could pave the way for more effective and safer treatments in the future by enabling the drug to be released more selectively in the cancerous area. Another factor is the rational design of combinations of plant extracts and anticancer drugs [128,129].

Such an approach will facilitate the transformation of hydrogel systems from mere carriers into programmable therapeutic platforms. Finally, to facilitate the translation of hydrogels containing plant extracts or plant-derived bioactive compounds into clinical practice, it is important to use more realistic biological models—such as 3D tumor models, organoids, and in vivo experiments—to ensure a realistic evaluation [130,131,132]. From a translational perspective, plant extract- or plant-derived bioactive compound-loaded hydrogel systems face additional regulatory and manufacturing barriers beyond biological validation. For botanical components, clinical development requires control of botanical raw materials, chemical and biological characterization, marker-compound quantification, batch-to-batch consistency, long-term formulation stability assessment, and GMP-compatible manufacturing [133]. For hydrogel-based systems, key translational challenges include sterilization without loss of bioactivity, reproducible loading and release behavior, long-term biodegradation, biosafety, manufacturing scalability, process validation, quality control, and scalable production [8,9,10]. Moreover, depending on composition and primary mode of action, these systems may be regulated as drug, device, or combination products, which can increase the complexity of preclinical and clinical evaluation [134]. Current clinical-trial records show that plant-derived compounds and related delivery approaches have been or are being investigated in cancer-related settings, including liposomal curcumin combined with radiotherapy and temozolomide in high-grade gliomas [135], inulin gel combined with ipilimumab and nivolumab in renal cell carcinoma [136], curcumin gel for radiation-induced oral mucositis [137], and plant exosome-mediated curcumin delivery to colon tissue and colon tumors [138]. However, these examples should be interpreted cautiously because they do not yet represent clinically validated plant extract- or plant-derived bioactive compound-loaded anticancer hydrogel systems. Therefore, most hydrogel systems discussed in this review should still be regarded as preclinical or proof-of-concept platforms rather than clinically validated anticancer treatments.

## 9. Conclusions

In cancer treatment, hydrogel systems containing plant extracts or plant-derived bioactive compounds represent a promising area of research for reducing the side effects of chemotherapeutic drugs, enhancing their efficacy, and enabling localized, controlled release. Studies have indicated that the use of phytochemicals such as curcumin, resveratrol, quercetin, mangiferin, limonin, EGCG, aloe vera components, and ginsenoside has been shown to provide multifaceted effects such as increased apoptosis, inhibition of proliferation, and regulation of oxidative stress; in particular, stimulus-responsive hydrogels are seen to offer significant advantages in terms of developing more selective delivery strategies. Local and postoperative hydrogel-based strategies have been explored in cancer-related settings such as breast cancer, colorectal cancer, glioblastoma, lung tumors, head and neck cancer, and peritoneal metastasis. Most of the existing systems still are in the in vitro, early preclinical, or proof-of-concept stage. Clinical translation of such systems requires not only demonstrating their in vivo efficacy but also standardizing their extract properties, ensuring batch-to-batch consistency in the composition of bioactive compounds, achieving GMP-compatible scalable manufacturing, and conducting long-term stability, sterility, and biosafety testing. Additionally, it involves clarifying regulatory requirements and validating pharmacokinetics, biodistribution, local retention, systemic toxicity, therapeutic efficacy, recurrence prevention, and patient safety. Hence, such systems cannot be treated as independent treatments; instead, they should be considered as well-designed therapeutic delivery systems, whose significance in the clinic will be decided upon the basis of their formulation, reproducible manufacture, stability, regulatory framework, and clinical validation.

## Figures and Tables

**Table 1:** Plant Extracts and Anticancer Bioactive Compounds.

Plant/Extract Source	Bioactive Group	Representative Bioactive Compound	Main Area of Use in the Literature	References
Curcuma longa (turmeric)/turmeric extract	Curcuminoid/polyphenolic compound	Curcumin	Widely used in cancer chemoprevention and preclinical anticancer mechanism studies, especially for proliferation, apoptosis, oxidative stress and angiogenesis.	[41,43,44,48]
*Mangifera indica* L. mango; leaves, bark, peel, kernel and seed-derived extracts	C-glucosyl xanthone/polyphenolic xanthonoid	Mangiferin	Used in preclinical anticancer and chemoprevention studies through modulation of oxidative stress, inflammation, apoptosis, cell-cycle regulation, angiogenesis, and metastasis-related pathways. Also explored in polymeric and nanotechnology-based delivery systems to address solubility and bioavailability limitations.	[51,52,56,59,60]
Vitis vinifera (grape skin/seed)	Stilbene/polyphenol	Resveratrol	Used in preclinical studies on proliferation, apoptosis, oxidative stress and metastasis-related pathways.	[41,43,44,48]
Camellia sinensis (green tea)	Catechin/flavanol/polyphenol	Epigallocatechin gallate (EGCG)	Used in chemoprevention and mechanistic studies focusing on antioxidant, pro-apoptotic and anti-angiogenic effects.	[48,49,50]
Onion, apple, grape, tea and various fruits/vegetables	Flavonoid/flavonol	Quercetin	Used in preclinical anticancer research on proliferation inhibition, apoptosis induction and oxidative stress modulation.	[41,43,44,48,49,50]
Glycine max (soybean)	Isoflavone	Genistein	Used in studies of hormone-related cancers and cancer-associated signaling pathways.	[41,43,44]
Parsley, celery, thyme, rosemary and selected vegetables	Flavonoid/flavone	Luteolin	Used in studies addressing inflammation, proliferation and apoptosis-related cancer mechanisms.	[41,43,44]
Capsicum species (chili pepper)	Capsaicinoid	Capsaicin	Used in cancer studies evaluating cell proliferation, apoptosis and oxidative stress responses.	[41,43,44]
Brassica vegetables, including broccoli, cabbage and cauliflower	Isothiocyanate/organosulfur compound	Sulforaphane	Used in chemoprevention studies related to detoxification enzymes, oxidative stress, apoptosis and cell-cycle regulation.	[41,43,44,57,58]
Berberis species, Coptis chinensis and Hydrastis canadensis	Isoquinoline alkaloid	Berberine	Used in preclinical studies on cancer cell proliferation, apoptosis and signaling pathway regulation.	[41,43,44,51]
Catharanthus roseus (Madagascar periwinkle)	Vinca alkaloid	Vincristine	Used as a clinically established plant-derived anticancer drug rather than a supportive dietary phytochemical.	[41,43,44]
Catharanthus roseus (Madagascar periwinkle)	Vinca alkaloid	Vinblastine	Used as a clinically established plant-derived anticancer drug in chemotherapy-based cancer treatment.	[41,43,44]
Taxus species (yew tree)	Taxane/terpenoid	Paclitaxel	Used as a clinically established plant-derived anticancer drug that acts mainly through microtubule stabilization.	[41,43,44,57]
Allium species, including garlic and onion	Organosulfur compounds	Allicin and diallyl sulfide derivatives	Used in chemoprevention-oriented studies on oxidative stress, inflammation and cancer cell death.	[42,57,58]
Tomato, carrot, leafy greens and colored fruits	Carotenoids	Lycopene, beta-carotene and lutein	Used in antioxidant and chemopreventive support studies; claims should remain compound- and context-specific.	[42,57]
Citrus fruits, mint, thyme, rosemary and aromatic plants	Terpenoids	Limonene, carvacrol and thymol	Used in preclinical research on bioactive plant secondary metabolites and selected anticancer mechanisms.	[42,57]

**Table 2:** Design of hydrogels incorporating bioactive agents and their applications in cancer treatment.

Plant Source/Bioactive Compound	Hydrogel System	Bioactive Compound Concentration/Dosage Range	Cancer Model/ Application	References
Curcumin and chrysin	Alginate–chitosan hydrogel	* NR	A549 lung cancer and T47D breast cancer cells	[83]
Curcumin	Graphene oxide-containing alginate hydrogel	2.5–7.5% *w*/*w* relative to alginate	Squamous cell carcinoma; local/topical cancer therapy	[84]
Curcumin modified with glycyrrhetinic acid	Glycyrrhetinic acid-modified curcumin supramolecular hydrogel	GA–Cur: 10 mg/mL	HepG2 hepatocellular carcinoma cells	[85]
Curcumin	Dialdehyde cellulose-crosslinked chitosan/ZnO nanocomposite hydrogel	Curcumin: 3–5 mg/mL; cell-treatment range: 7.8–1000 μg/mL	A431 human skin carcinoma cells; enhanced curcumin delivery and anticancer bioactivity	[86]
Resveratrol	Resveratrol nanoemulsion-loaded thermosensitive hydrogel	Resveratrol: 25 mg/mL; cell-treatment range: 20–40 μM	MCF-7 breast cancer cells	[87]
Resveratrol	Click-crosslinked hyaluronic acid hydrogel	Resveratrol: ≈5 mg/mL in hydrogel	Triple-negative breast cancer; intratumoral application	[88]
Grape seed-derived oligomeric proanthocyanidins	OPC-containing smart hydrogel scaffold	OPC: 2, 4, and 6 wt%	Melanoma treatment and wound healing	[89]
Green tea extract and curcumin	Gellan gum/chitosan bilayer scaffold	Green tea extract: 2 wt%; curcumin: 1 wt%	MCF-7 breast cancer cells	[90]
EGCG	EGCG, indocyanine green, and gold nanoparticle-containing smart hydrogel	EGCG: 2 mg/mL in hydrogel; 40 μg/mL in vitro; 10 mg/kg in vivo	Breast cancer; multimodal therapy	[91]
Aloe vera	Aloe–alginate hydrogel	Aloe vera: 50–67 wt% in aloe–alginate hydrogel	Cervical cancer cells	[92]
Aloe vera gel powder	Alginate/Aloe vera/chitosan hydrogel	Aloe vera gel powder: 1% solution	MDA-MB-231 breast cancer and U87MG glioblastoma spheroids	[93]
Onion peel-derived quercetin	Chitosan–cellulose/ZnO nanohybrid hydrogel	Quercetin: 0.5–2.0 mg/mL; cell-treatment range: 7.8–1000 μg/mL	L929 fibroblasts and A431 skin carcinoma cells	[94]
Quercetin and taxifolin	pH-responsive injectable hydrogel	Quercetin: 26 μM; taxifolin: 30 μM	MDA-MB-231 and MDA-MB-468 triple-negative breast cancer cells	[95]
Quercetin	Xanthan gum/guar gum/halloysite nanotube hydrogel	Quercetin: 5 μg/mL	HepG2 liver cancer cells	[96]
*Clitoria ternatea* plant extract-derived biogenic silver nanoparticles	Sodium alginate/gelatin hydrogel beads containing Ag@CT nanoparticles	Ag@CT nanoparticles: 2% in hydrogel beads; cell-treatment range: 0–100 μg/mL	A549 lung cancer cells; anticancer and apoptosis assessment	[97]
Naringenin	Chitosan-based ZnO nanohybrid hydrogel	Naringenin: 0.5–2.0 mg/mL; cell-treatment range: 7.8–1000 μg/mL	A431 skin carcinoma cells	[98]
Naringenin nanocrystals	Pluronic F127 hydrogel	Naringenin: 16 mg; cell-treatment range: 50–350 μM	Skin cancer; topical delivery	[99]
Naringenin	pH-responsive dual-layered nanohydrogel based on protein–polysaccharide complexes	Naringenin: 12–60 mg/mL	Colorectal cancer-targeted delivery	[100]
Mangiferin	RADA16-I self-assembling peptide-based in situ hydrogel	Mangiferin: 0.3 mg/mL	KYSE30 and DLD-1 tumor cells; controlled release, enhanced proliferation inhibition, and reduced toxicity toward 293T normal renal epithelial cells	[101]
Mangiferin	Phospholipid-based topical nano-hydrogel	Mangiferin: 2% *w*/*w*, equivalent to 0.02 g/g gel	MCF-7 breast cancer cells; controlled release, enhanced cellular uptake, improved skin retention, and increased in vitro anticancer activity	[102]
*Parthenocissus quinquefolia* L. extract	pH-responsive antioxidant-biodegradable hydrogel using plant extract as crosslinker	* NR	Release assessment and anticancer effect	[62]
Rutin	Antioxidant-biodegradable poly(acrylic acid-co-2-hydroxyethyl methacrylate) hydrogel using rutin as crosslinker	* NR	Drug release and anticancer activity	[63]

* NR: Bioactive compound concentration/dosage range was not reported.

**Table 3:** Hydrogels Containing Bioactive Compounds and Anticancer Drugs in the Literature.

Bioactive Compound/Plant-Derived Component	Chemotherapeutic Drug	Bioactive Compound Concentration/Dosage Range	Chemotherapeutic Drug Concentration/Dosage Range	Hydrogel/ Nanogel System	Cancer Model/ Application	References
Aloe vera	Imatinib	Aloe vera: 20% in SA/PVA/AV hydrogel	Imatinib: 25 µM	Sodium alginate/PVA/Aloe vera hydrogel	Breast cancer treatment	[105]
Aloe vera	Doxorubicin	Aloe vera: NR	Doxorubicin: 5 mg/mL	Doxorubicin-loaded bacterial ghosts embedded in natural hydrogels, including Aloe vera hydrogel	Cancer drug delivery/3D culture-oriented release system	[106]
Curcumin	Doxorubicin	Curcumin: 1.0 mg per hydrogel	Doxorubicin: 1.0 mg per hydrogel	pH-sensitive injectable in situ hydrogel composed of chitosan, graphene, and cellulose nanowhisker	Cancer combination therapy	[107]
Curcumin	Doxorubicin	Curcumin: 100–200 µM	Doxorubicin: 50–100 µM; Cur/Dox combinations	Temperature- and pH-responsive injectable chitosan hydrogel	Solid tumor treatment/long-lasting local release	[108]
Curcumin	Doxorubicin	Curcumin: 0.1–100 µM; combination hydrogel: 1–15 µM	Doxorubicin: 0.05–10 µM; combination hydrogel: 0.05–0.6 µM	Self-assembling peptide hydrogel	Head and neck cancer	[109]
Curcumin	Doxorubicin	Curcumin: 1.82 mg/mL encapsulated in Fmoc-FF nanogels	Doxorubicin: NR	Fmoc-FF peptide nanogel	Thyroid cancer cells; nanogel-mediated delivery, sustained curcumin release, cellular internalization, and delayed nuclear uptake of doxorubicin	[110]
Quercetin	Doxorubicin	Quercetin: 250 µg/mL	Doxorubicin: 10 mg in hydrogel	Gelatin–oxidized alginate hydrogel with quercetin-loaded chitosan-coated zein nanoparticles	Localized breast cancer therapy	[111]
Limonin	Doxorubicin	Limonin: 25 mg/L in vitro; 25 mg/kg in vivo	Doxorubicin: 9 mg/L in vitro; 9 mg/kg in vivo	Pectin-based self-healing hydrogel with covalently coupled doxorubicin and limonin loading	Lung tumor therapy	[112]
Resveratrol	Doxorubicin	Resveratrol: 324 µg/mL	Doxorubicin: 516 µg/mL	Chitosan/albumin/hydroxypropyl-β-cyclodextrin composite nanogel	Reduction in doxorubicin-related cardio-/neurotoxicity; anticancer drug delivery	[113]
Curcumin	Paclitaxel	Curcumin: 2–10% *w*/*w*	Paclitaxel: 2 wt% relative to Fud-F127	Fucoidan–Pluronic F127 nanogel	Synergistic breast cancer treatment	[114]
Curcumin	Paclitaxel	Curcumin: NR	Paclitaxel: NR	Heparin–Poloxamer P403 hybrid nanogel	Breast cancer	[115]
Curcuminoid	Cisplatin hydrate	Curcuminoid: 4.4%	Cisplatin hydrate: 22.3% loading	Heparin–Poloxamer P403 nanogel	Antitumor activity	[116]
Curcumin	Lauroyl-gemcitabine/GemC12	Curcumin: 3.1 ± 0.4 mg/mL in GemC12-Cur-LNC	GemC12: 18.9 ± 1.7 mg/mL in GemC12-Cur-LNC;	GemC12 lipid nanocapsule hydrogel	Glioblastoma and glioma stem-like cells	[117]
Curcumin	Oxaliplatin	Curcumin: 16 µM in vitro; 5 mg/kg in vivo	Oxaliplatin: 16 µM in vitro; 5 mg/kg in vivo	Sprayed hyaluronic acid-based multidrug composite hydrogel	Postoperative colorectal cancer and peritoneal metastasis prevention	[118]
Curcumin	5-Fluorouracil	Curcumin: 1–2 mg/mL; 5-FU/CUR ratios: 0.5–1.5:1–2, mg/mL	5-Fluorouracil: 0.5–1.5 mg/mL	Silk fibroin hydrogel	Adjuvant therapy in colorectal cancer	[119]
Curcumin	5-Fluorouracil	Curcumin: micelle/Cur ratios 90–85:5–20 mg/mL	5-Fluorouracil: 1–25 mg/mL	Schiff base-crosslinked injectable hydrogel using Pluronic F127 micelles for curcumin and hydrogel network for 5-FU	HT-29 colorectal cancer cells/colorectal cancer combination therapy	[120]
Curcumin	5-Fluorouracil	Curcumin: micelle/Cur weight ratio 85:15 mg/mL	5-Fluorouracil: 1 mg/mL	Gelatin-based injectable hydrogel/microgel composite	Local synergistic therapy of colorectal cancer	[121]
Pectin	Doxorubicin	Pectin: 50 mg in hydrogel formulation	Doxorubicin: 50 mg/L	Poly(3-sulfopropyl acrylate)/pectin hydrogel functionalized with silver and quantum dots	Sustained doxorubicin delivery; plant-derived polysaccharide-based supportive hydrogel	[122]

**Table 4:** Distribution of hydrogel-based delivery strategies across major cancer types in the reviewed literature.

Cancer Type	Application Coverage in the Reviewed Hydrogel Studies	Hydrogel-Based Delivery Strategy	Therapeutic Rationale
Breast cancer	High	Injectable, thermosensitive, bilayer scaffold, and nanogel-based systems	Local retention, sustained release, and combination therapy
Colorectal cancer	High	pH-responsive, postoperative local, and injectable hydrogel systems	Local delivery, recurrence control, and pH-responsive drug release
Lung cancer	High	Alginate-based hydrogels, hydrogel beads, and self-healing hydrogel systems	Controlled release, toxicity reduction, and experimental model suitability
Skin cancer/ melanoma	Moderate	Topical, local, photothermal, and nanocomposite hydrogel systems	Anatomical accessibility and feasibility of localized treatment
Liver cancer	Moderate	pH-responsive and targeted hydrogel systems	Delivery of poorly soluble compounds and tumor-associated release
Cervical cancer	Low	Local hydrogel systems	Potential for local delivery
Glioblastoma	Low	Post-surgical local delivery and nanocapsule-hydrogel systems	Local retention and recurrence control
Head and neck cancer	Low	Self-assembling peptide hydrogel systems	Sustained local release
Prostate cancer	Very low/not prominent	Not clearly established in the reviewed studies	Insufficient evidence on plant-derived hydrogel systems
Stomach cancer	Very low/not prominent	Not clearly established in the reviewed studies	Limited development of localized hydrogel strategies

This review is based on studies of hydrogels loaded with plant extracts and plant-derived bioactive compounds, which are discussed in Section 6 and Section 7 of this review. Detailed references are provided in the relevant application and combination tables.

## Data Availability

Data availability is not applicable to this article as no new data were created or analyzed in this study.
